# D-Allulose Ameliorates Dysregulated Macrophage Function and Mitochondrial NADH Homeostasis, Mitigating Obesity-Induced Insulin Resistance

**DOI:** 10.3390/nu15194218

**Published:** 2023-09-29

**Authors:** Heekyong R. Bae, Su-Kyung Shin, Youngji Han, Ji-Hyeon Yoo, Suntae Kim, Howard A. Young, Eun-Young Kwon

**Affiliations:** 1 Department of Food Science and Nutrition, Kyungpook National University, Daegu 41566, Republic of Korea; 2 Center for Food and Nutritional Genomics, Kyungpook National University, Daegu 41566, Republic of Korea; 3 Omixplus, LLC., Gaithersburg, MD 20850, USA; 4 Cancer Innovation Laboratory, Center for Cancer Research, National Cancer Institute, Frederick, MD 21702, USA; younghow@mail.nih.gov; 5 Center for Beautiful Aging, Kyungpook National University, Daegu 41566, Republic of Korea

**Keywords:** allulose, obesity, insulin resistance, type 2 diabetes, NADH homeostasis, mitochondrial translation

## Abstract

D-allulose, a rare sugar, has been proposed to have potential benefits in addressing metabolic disorders such as obesity and type 2 diabetes (T2D). However, the precise mechanisms underlying these effects remain poorly understood. We aimed to elucidate the mechanisms by which D-allulose influences obesity-induced insulin resistance. We conducted gene set enrichment analysis on the liver and white adipose tissue of mice exposed to a high-fat diet (HFD) along with the white adipose tissue of individuals with obesity. Our study revealed that D-allulose effectively suppressed IFN-γ, restored chemokine signaling, and enhanced macrophage function in the livers of HFD-fed mice. This implies that D-allulose curtails liver inflammation, alleviating insulin resistance and subsequently impacting adipose tissue. Furthermore, D-allulose supplementation improved mitochondrial NADH homeostasis and translation in both the liver and white adipose tissue of HFD-fed mice. Notably, we observed decreased NADH homeostasis and mitochondrial translation in the omental tissue of insulin-resistant obese subjects compared to their insulin-sensitive counterparts. Taken together, these results suggest that supplementation with allulose improves obesity-induced insulin resistance by mitigating the disruptions in macrophage and mitochondrial function. Furthermore, our data reinforce the crucial role that mitochondrial energy expenditure plays in the development of insulin resistance triggered by obesity.

## 1. Introduction

D-allulose (referred to as allulose hereafter), also known as psicose, has garnered significant attention as an innovative sugar substitute in recent years. It shares a molecular formula with fructose, making it the closest natural sweetener to fructose in terms of taste, yet it contains almost zero calories compared to sucrose [1,2]. In 2012, the US Food and Drug Administration granted approval for the safety of allulose (GRN No. 400). Other countries, including Japan, Singapore, and South Korea, have also granted their approvals for its safety. Despite its relative stability as a naturally occurring substance in foods like figs, kiwis, and raisins, allulose still requires advancements in mass production techniques to make it more widely accessible and usable. Fortunately, recent advancements in technology for allulose production are progressing rapidly [3,4], raising high expectations for its future widespread use.

We have reported that allulose exhibits significant physiological effects, including the normalization of body weight and fat-pad mass in obese animals under isocaloric conditions, as well as anti-hyperlipidemic, anti-hyperglycemic, and anti-diabetic properties [5,6,7]. Recent clinical studies have indicated that it can reduce postprandial blood glucose levels, can improve insulin resistance, and has potential benefits in managing obesity and type 2 diabetes (T2D) [8,9,10,11]. These effects stem from its unique metabolic properties, positioning it as a promising candidate for use as both a sugar substitute and a dietary supplement.

Additionally, recent animal studies have revealed that allulose influences metabolic physiology by modulating appetite, promoting glucagon-like peptide-1 (GLP-1) release through vagal afferents [12,13], and facilitating hepatic glucokinase translocation [14]. These mechanisms collectively result in reduced food intake and enhanced glucose tolerance. Furthermore, allulose exerts its influence on central neurons, including those responsive to GLP-1 and proopiomelanocortin (POMC) neurons, leading to decreased food consumption when centrally administered in mice [15]. The convergence of these diverse physiological effects underscores the potential of allulose as an alternative sweetener for addressing obesity and diabetes.

Obesity is a metabolic disorder characterized by excessive fat accumulation and is closely associated with chronic inflammation. In obesity, there is a sustained low-grade inflammatory response within adipose tissue, involving various inflammatory factors and cells [16]. This chronic inflammation is closely linked to the development of obesity-related complications and plays a significant role in the progression of various metabolic disorders like T2D [17]. Understanding the mechanisms by which obesity triggers chronic inflammation holds promising implications for the development of novel therapeutic strategies aimed at mitigating obesity-related metabolic disorders.

As recently highlighted in our report, we demonstrated in high-fat-diet mice that early immune responses within the liver play a significant role in immune cell infiltration within adipose tissue and systemic chronic inflammation [18]. Based on these findings and research methodologies, we aim to investigate the mechanisms through which allulose can potentially mitigate chronic inflammation induced by obesity. Through this approach, we aim to elucidate the key mechanisms through which obesity may contribute to the development of T2D.

## 2. Materials and Methods

### 2.1. Animals and Diet

In this study, we utilized our experimental data that have been deposited in the Gene Expression Omnibus (GEO) database with accession number GSE137365. Briefly, a total of 27 male C57BL/6J mice (4 weeks old) were purchased from the Jackson Laboratory and were fed a pelletized commercial non-purified diet for 1 week after arrival. They were then randomly divided into three groups (n = 9) and fed the respective experimental diets for 16 weeks. The HFD group was fed a semi-purified diet with 39.5% of the total energy from fat, which was achieved by replacing carbohydrate energy with lard and corn oil, and which had the same amounts of vitamins and minerals per kJ as control littermates. The diets used in the study included a normal diet (ND) control (based on the American Institute of Nutrition AIN-76 semisynthetic diet), an HFD group (which contained 20% fat and 1% cholesterol based on the AIN-76 diet), and a diet with 5% D-allulose (referred to as ALLU), where sucrose in the HFD was substituted with 5% D-allulose (*w*/*w*). Of note, since the physiological data at 16 weeks in GSE137365 closely correspond with the data at 24 weeks in GSE39549, emphasizing the comparison of phenotypes becomes more crucial than solely comparing age for accurate data integration.

### 2.2. mRNA Sequencing and Data Processing

As mentioned above, we employed our experimental data deposited in GSE137365. Briefly, total RNA from the liver and epididymal white adipose tissue (eWAT) (n = 9 per group) was extracted using the TRIzol method and evaluated for quality and quantity with an Agilent 2100 bioanalyzer RNA kit. An RNA sequencing library was prepared with an Illumina TruSeq Stranded mRNA Sample Preparation kit and assessed with the Agilent 2100 bioanalyzer DNA kit. The libraries were sequenced on an Illumina NextSeq500 sequencer and quality checked with the FastQC tool (http://www.bioinformatics.babraham.ac.uk/projects/fastqc, accessed on 6 September 2023). The raw reads were trimmed using the cutadapt software [19] and mapped to the mouse reference genome mm9 of the UCSC genome using the STAR software [20]. Differential gene expression analysis was performed with the cuffdiff software in the cufflinks package. The statistical significance of differential gene expression was determined using a false discovery rate (FDR) adjusted *p*-value threshold of 0.05. When necessary, the significantly differentially expressed genes were further determined using a log2 fold-change threshold of 1 or greater.

### 2.3. GSEA Pre-Ranked Analysis

The gene set enrichment analysis (GSEA) pre-ranked analysis method is a variation of GSEA in which gene expression profiles are pre-ordered based on the direction and degree of correlation among each gene and the pathway of interest [21]. Initially, we ranked the gene expression data according to the fold change in both the liver and eWAT at each age. The algorithm of the GSEA pre-ranked analysis method computes the normalized enrichment scores (NES) and enrichment score (ES) using a pre-ordered gene list through 1000 permutations. We considered significance when the p-adjusted value was less than 0.05 and the false discovery rate (FDR) was less than 0.05. To discover enriched pathways of interest, we employed annotated gene collections acquired from the Molecular Signatures Database (MSigDB v7.4), particularly focusing on hallmark gene sets and other publicly available databases such as Mouse Genome Informatics (MGI).

### 2.4. Patient Data Acquisition and Processing

To validate our findings using human datasets, we employed the GEO database and conducted a comparative analysis of the gene expression profiles between insulin-resistant and insulin-sensitive patients in omental adipose tissue derived from morbidly obese individuals (GSE20950). As described in a previous report [22], the body mass index (BMI) was carefully matched between the two groups of morbidly obese individuals. The insulin-resistant group included 10 subjects, consisting of 6 women and 4 men, while the insulin-sensitive group comprised 10 subjects, with 8 women and 2 men. The age range of the participants spanned from 31 to 57 years. The Affymetrix Human Genome 133 Plus 2.0 Array (platform: GPL570) was employed for gene expression profiling in this investigation. To perform the differential expression analysis, we utilized Gene Expression Omnibus 2R (GEO2R), which is an interactive web tool provided by the National Center for Biotechnology Information (NCBI). GEO2R utilizes the *t*-test as the default statistical method for comparing the means of two groups.

### 2.5. Pathway Analysis and Data Visualization

In this study, we set the level of statistical significance at *p* < 0.05. To identify enriched genes in the liver and eWAT, we used fold-change values with a consistent cutoff value. For example, a positive enrichment fold-change cutoff value of 1.0 was applied in the HFD group, while a negative enrichment cutoff value of −1.0 was used for allulose treatment. For pathway analysis based on a variety of functional genomics databases, we employed Enrichr, a web-based gene enrichment analysis tool, to confirm the notable correlation between the chosen list of genes and a particular gene. To identify network interactions among the enriched genes and pathways, we used the GeneMANIA plugin in CytoScape 3.9.1 [23]. The enriched genes from the previous step were entered into GeneMANIA, which predicted a range of interaction types, including physical interactions, co-expression, co-localization, and pathway sharing. The resulting network was visualized using CytoScape 3.9.1, and hub genes were identified based on their degree of connectivity within the network. To generate data visualizations using R, we utilized ggplot2 in R to create data visualizations such as scatterplots, boxplots, and heatmaps and customized the appearance of the plots with built-in themes and scales, including font sizes, colors, and axis labels. We also utilized the MultiExperiment Viewer (MeV 4.9.0) software to create a heatmap displaying the pattern of gene expression.

## 3. Results

### 3.1. Time-Dependent Hallmark Gene Set Analysis in the Liver and eWAT from HFD Mice

**Figure 1:**
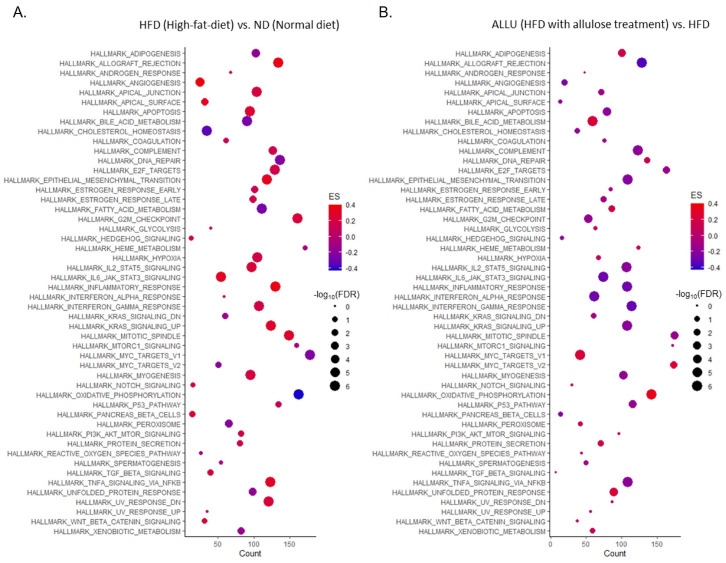
Hallmark gene set analysis in the liver from mice fed the HFD with or without allulose treatment. Enrichment analysis was performed using fifty hallmark gene sets from the MSigDB. The x-axis represents the count of genes that exhibited significant enrichment within each pathway, determined using the GSEA algorithm. The y-axis displays fifty hallmark pathways. The size of the dots reflects the significance based on −log10(FDR *q*-value), and the color of the dots indicates the ES, with red representing a positive score and blue representing a negative score. Panel (**A**) shows a dot plot of 50 hallmark gene sets analyzing the response to HFD, while panel (**B**) shows a dot plot of 50 hallmark gene sets analyzing the response to HFD with allulose treatment.

**Figure 2:**
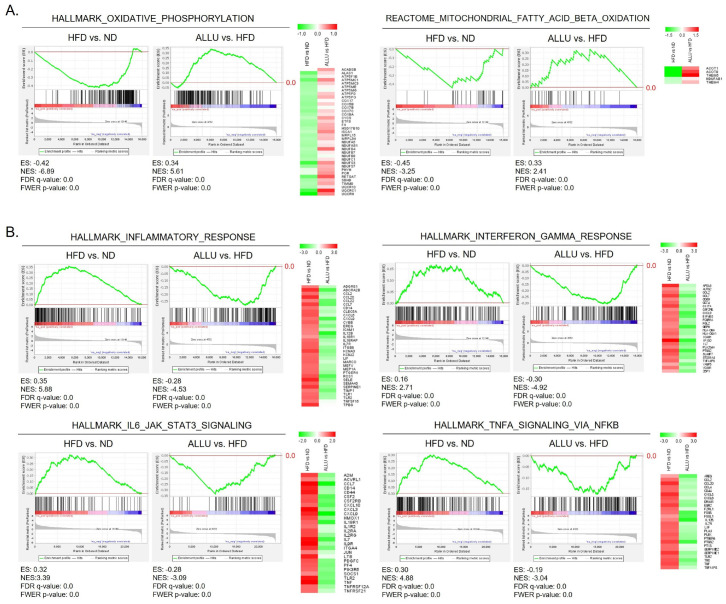
Effects of allulose treatment on inflammatory responses and lipid metabolism in the liver of HFD-fed mice. (**A**) Enrichment plots showing suppression of lipid metabolism in the liver of HFD-fed mice and its restoration upon allulose treatment. Gene sets related to oxidative phosphorylation, adipogenesis, bile acid metabolism, and fatty acid metabolism showed negative enrichment in HFD mice compared to normal diet, while allulose treatment led to positive enrichment. (**B**) Enrichment plots showing induction of inflammatory responses in the liver of HFD-fed mice and its attenuation upon allulose treatment. The baseline (0.0) of the enrichment score is indicated with a red color line. Heatmaps display the expression levels of highly enriched genes within each gene set, comparing HFD-fed mice with and without allulose treatment.

We conducted 50 hallmark gene set analyses from the MSigDB in GSEA to characterize the overall responses of allulose treatment to an HFD in the liver and eWAT. Figure 1 presents the analysis results between the HFD vs. HFD with allulose treatment (referred as ALLU) in the liver. Our findings indicated that the allulose treatment resulted in the opposite regulation of gene sets that were significantly altered by HFD. Specifically, among the 50 hallmark gene sets, those involved in lipid metabolism and immune responses were specifically affected by the HFD and are demonstrated in detail in Figure 2. As shown in Figure 2A, the HFD led to a negative enrichment of gene sets related to oxidative phosphorylation and mitochondrial fatty acid beta-oxidation. Conversely, the allulose treatment in the HFD mice resulted in the positive enrichment of these gene sets. The enriched genes, which were selected based on the top changes, are listed beside each enrichment plot generated from GSEA.

As for immune responses (Figure 2B), the HFD led to a positive enrichment of gene sets related to an inflammatory response, IFN-γ response, IL-6_JAK_STAT3 signaling, and TNF-α signaling via NF-κB. The allulose treatment significantly induced a negative enrichment of these gene sets. In eWAT, parallel to the observations in the liver, the allulose treatment yielded consistent results, as illustrated in Supplemental Figure S2.

### 3.2. Suppression of Chemokine Expression and Dysregulated Macrophage Function by Allulose in the Liver and eWAT from HFD Mice

**Figure 3:**
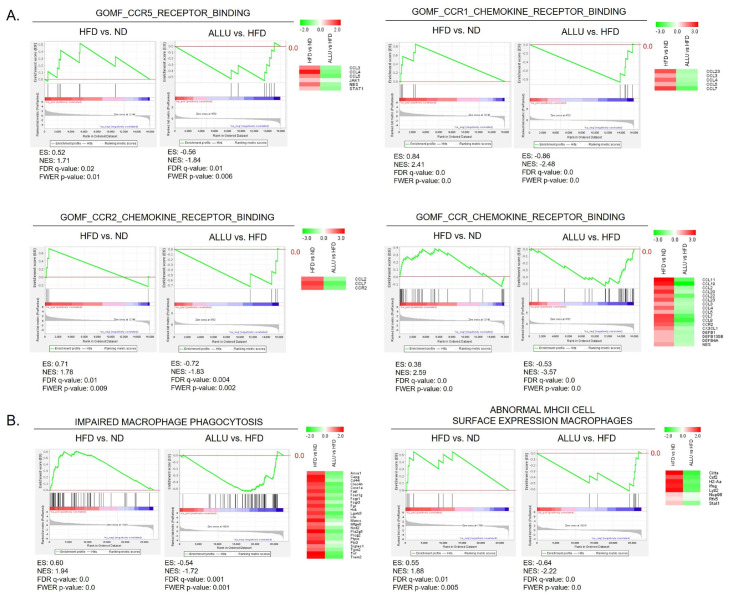
Effects of allulose treatment on chemokine receptor signaling and macrophage dysfunction in the liver of HFD-fed mice. (**A**) Plots depicting positive enrichment generated by GSEA in response to HFD and its reversal upon allulose treatment with gene sets of CCR1, CCR2, CCR5, and overall CCR chemokine receptor binding derived from the GOMF database. (**B**) Positively enriched plots generated from GSEA in response to HFD and its reversal upon allulose treatment with gene sets of impaired antigen-specific response and impaired oxidative burst derived from Human Phenotype (HP) and Geon Ontology Biological Process (GOBP) databases and gene sets of impaired macrophage phagocytosis, and abnormal MHC II cell surface expression on macrophages derived from Mouse Genome Informatics (MGI) database in HFD-fed mice liver. A heatmap illustrates the expression levels of the most enriched genes in these targeted gene sets, comparing HFD-fed mice with and without allulose treatment.

We previously reported that dysregulated macrophage function via the chronic expression of IFN-γ is crucial for HFD-induced chronic inflammation [24,25]. To further investigate this, we examined the expressions of various chemokine receptors, which included the CCR1, CCR2, CCR3, CCR4, CCR5, and CCR6 pathways. Our results demonstrated that the HFD notably activated the CCR1, CCR2, and CCR5 pathways in both the liver and eWAT, as depicted in Figure 3A and Supplemental Figure S3. As demonstrated in Figure 3A, our findings indicated that in the liver, the HFD resulted in a positive enrichment of gene sets related to CCR1, CCR2, and CCR5 chemokine receptor binding. The gene set analysis for the overall CCR chemokine receptor binding indicated that the HFD significantly induced the expression of CCL11, CCL19, CCL20, and CCL8. Conversely, the treatment with allulose suppressed their expression. These findings suggest that besides macrophages, other immune cells, such as dendritic cells and T cells, could be recruited by an HFD and contribute to HFD-induced pathogenesis. The heatmap beside each enrichment plot generated from GSEA demonstrates enriched genes in the CCR1, CCR2, and CCR5 responses as well as the total CCR in the liver and eWAT.

Since the activation of CCR1, CCR2, and CCR5 receptors is known to be involved in the recruitment of innate immune cells such as macrophages and monocytes [26,27], we further characterized the abnormal macrophage function. As demonstrated in Figure 3B, the HFD positively enriched gene sets presenting abnormal macrophage function such as impaired macrophage phagocytosis and abnormal MHC II cell surface expression on macrophages. The treatment with allulose to the HFD-fed mice resulted in the reversal of these gene sets, as seen in the negative enrichment of impaired macrophage phagocytosis and abnormal major histocompatibility complex (MHC) II cell surface expression on macrophages. The pathway analysis of the significantly enriched genes from listed gene sets related to abnormal macrophage function revealed that IFN-γ signaling had the highest association (Supplemental Figure S4A). Additionally, our observations revealed that the HFD induced an increase in anti-inflammatory responses, particularly interleukin-10 (IL-10) signaling, which may serve as a counterbalance to maintain homeostasis between pro- and anti-inflammatory states. However, the allulose treatment exhibited a suppressive effect on IL-10 signaling as well (Supplemental Figure S4B), indicating its direct inhibitory impact on M1 macrophage activation.

### 3.3. Correlation between Mitochondrial Energy Expenditure and mRNA Translation Processes in the PBMCs from T2D Patients

**Figure 4:**
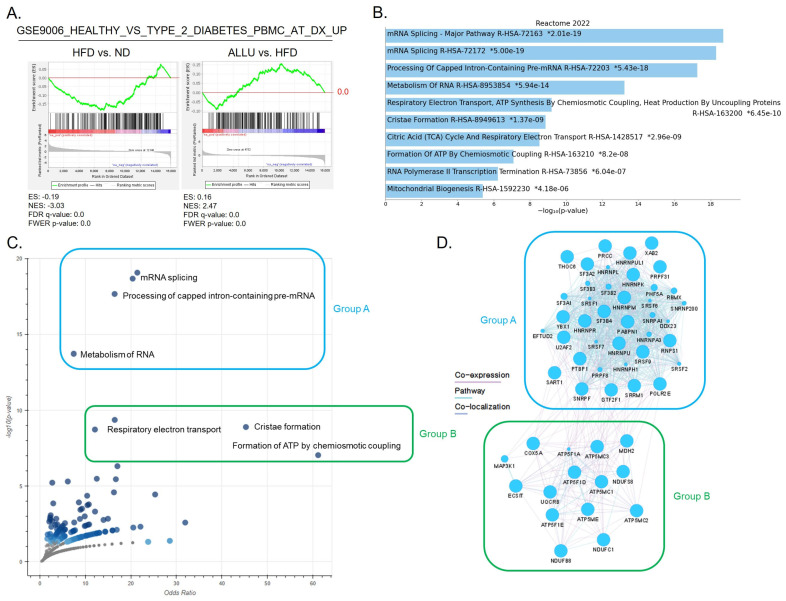
Comparison analysis of PBMC gene expression profiles in healthy donors and T2D Patients, with enriched genes by allulose treatment in HFD mice. (**A**) Enriched plots generated by GSEA showing the effect of the allulose treatment on the gene set of GSE9006 healthy vs. T2D PBMCs (upregulated genes from healthy donors compared to T2D at the time of diagnosis). (**B**) Enrichr pathway analysis showing the top 10 pathways of enriched genes in (**A**) based on Reactome 2022 database. The x-axis represents the significance based on −log10(*p*-value), with the top pathways listed in descending order. (**C**) A volcano plot depicting the top 10 pathways from (**B**), with the y-axis representing −log10(*p*-value) and the x-axis representing the odds ratio. Based on their characteristics, the pathways were categorized into two groups. Group A (represented by a blue square) includes mRNA translation pathways, while Group B (represented by a green square) consists of mitochondrial energy expenditure pathways. (**D**) GeneMANIA network analysis was performed on two groups from (**C**) using CytoScape 3.9.1. Interactions are depicted by purple lines (co-expression), light blue lines (pathway), and blue lines (co-localization).

Considering that immune cells, particularly macrophages, are the key factors in the development of HFD-induced chronic inflammation and T2D, we examined the gene expression profiles of peripheral blood mononuclear cells (PBMCs) obtained from patients diagnosed with T2D. In Figure 4A, it is demonstrated that the allulose treatment group exhibited an opposing response to the HFD group in the gene set that was upregulated when comparing PBMCs from healthy donors with those from patients diagnosed with T2D at the time of diagnosis. We then identified the enriched genes in both groups and performed a pathway analysis using the Enrichr algorithm. Figure 4B displays the top 10 pathways of enriched genes that were oppositely regulated between the HFD and allulose treatment groups, which included mRNA splicing, the processing of capped intron-containing pre-mRNA, the metabolism of RNA, respiratory electron transport, cristae formation, and the formation of ATP by chemiosmotic coupling. We classified these pathways into two groups (Group A and Group B) based on −log10 (*p*-value) and odds ratio, as shown in Figure 4C, where Group A represents mRNA translation pathways and Group B represents mitochondrial energy expenditure pathways. In Figure 4D, we present evidence of co-expression between the genes in Group A and Group B, indicating an interactive regulation of both the mitochondrial energy expenditure and mRNA translation processes.

### 3.4. Effect of Allulose on RBPs and Its Potential Implications for Hepatic Insulin Resistance Triggered by HFD

**Figure 5:**
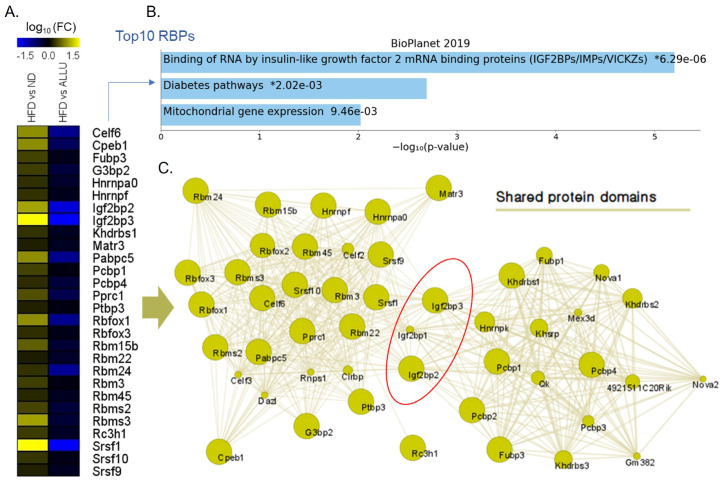
Comparison analysis of RNA-binding proteins, with enriched genes by allulose treatment in HFD mice. (**A**) Heatmap showing RBPs that were significantly increased in response to HFD and decreased by the allulose treatment. (**B**) Enrichr pathway analysis showing the significantly changed pathways of top 10 enriched RBPs in (**A**) based on the BioPlanet 2019 database. (**C**) Gene clustering analysis of the significantly enriched RBPs in (**A**) using the GeneMANIA plugin in Cytoscape 3.9.1. Interactions between the RBPs are depicted by yellow lines, indicating shared protein domains.

In order to investigate the involvement of mRNA translation in T2D related to HFD, we employed the RBPmap database, a web server designed for mapping RNA-binding protein (RBP) binding sites [28]. We conducted a comprehensive analysis by comparing the complete set of 132 RBPs with human and mouse motifs against the dataset associated with allulose treatment. As shown in Figure 5A, we selected RBPs that were significantly increased in response to HFD and confirmed their decrease in the allulose treatment group. Among these genes, we performed the pathway analysis on the top 10 significantly changed genes based on the Enrichr algorithm (Figure 5B). The top three pathways detected according to the BioPlanet 2019 database were the binding of RNA by insulin-like growth factor 2 mRNA-binding proteins (IGF2BPs), diabetes pathways, and mitochondrial gene expression. Gene clustering using Cytoscape 3.9.1 of the significantly increased RBPs in the HFD showed that they formed two large clusters, which were connected by shared protein domains of Igf2bp2 and Igf2bp3 (Figure 5C). This finding suggests that allulose treatment may regulate cytoplasmic and mitochondrial mRNA translation by targeting RBPs, whose expression is linked to the development of T2D.

### 3.5. Effect of Allulose on Altered Mitochondrial NADH Homeostasis and Mitochondrial Translation in the Liver and eWAT from HFD Mice

**Figure 6:**
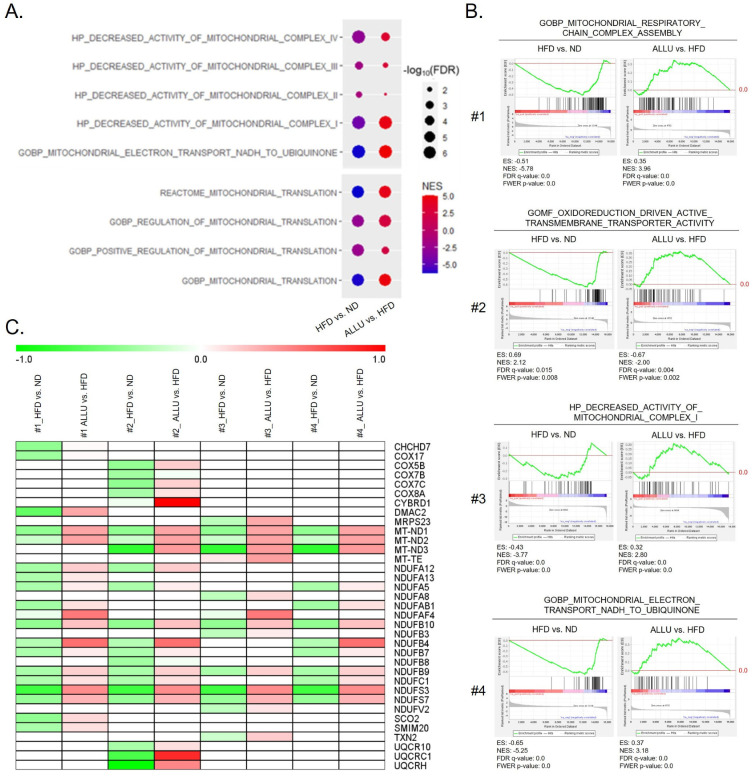
Effects of allulose treatment on mitochondrial energy expenditure and mRNA translation in the liver of HFD-fed mice. (**A**) Dot plots representing gene sets with significant enrichment associated with the activity of mitochondrial complexes and regulation of the mitochondrial translation process. The y-axis shows gene sets that include mitochondrial complex I, II, III, and IV activities and mitochondrial electron transport from NADH to ubiquinone from Reactome, HP, and GOBP. The groups in the x-axis indicate the response of HFD with or without allulose treatment. The dot size represents significance according to −log10(FDR *q*-value) and the dot color indicates NES. (**B**) Enriched plots generated from GSEA showing suppression of mitochondrial energy expenditure procedure in HFD-fed mice liver and its reversal upon allulose treatment. Gene sets include mitochondrial respiratory chain complex assembly, oxidoreduction-driven active transmembrane transporter activity, decreased activity of mitochondrial complex I, and mitochondrial electron transport from NADH to ubiquinone derived from GOBP, Gene Ontology Molecular Function (GOMF), and HP databases. (**C**) Heatmap of representative enriched genes from gene sets (#1–#4) in (**B**) with or without allulose treatment.

Next, we conducted a targeted analysis to examine gene sets associated with mitochondrial energy expenditure and mitochondrial translation processes. Figure 6A shows that the HFD negatively affected gene sets related to mitochondrial complexes, which were regulated in the opposite direction by the allulose treatment. A heatmap illustrates the expression levels of the most enriched genes in these targeted gene sets, comparing HFD-fed mice with and without allulose treatment. The allulose treatment strongly reversed the downregulation of mitochondrial electron transport from NADH to ubiquinone in complex I caused by the HFD. Likewise, mitochondrial translation and its regulation were downregulated by the HFD and significantly reversed by the allulose treatment. Figure 6B shows that gene sets involved in mitochondrial respiratory chain complex assembly and oxidoreduction-driven active transmembrane activity were strongly downregulated by the HFD, which was consistent with the downregulation of gene sets related to a decreased activity of mitochondrial complex I and mitochondrial transport from NADH to ubiquinone shown in Figure 6A. The left panel in each gene set shows negative enrichment in response to the HFD compared to the ND, and the right panel shows positive enrichment upon allulose treatment in the HFD-fed mice. The heatmap shown in Figure 6C lists the genes from these gene sets for each group (#1 to #4 in Figure 6B) with or without allulose treatment. These patterns were similarly detectable in eWAT, as shown in Supplemental Figure S5.

### 3.6. Alteration of Mitochondrial NADH Homeostasis and Mitochondrial Translation in the Omental Adipose Tissue Obtained from Human Obese Subjects with Insulin Resistance

**Figure 7:**
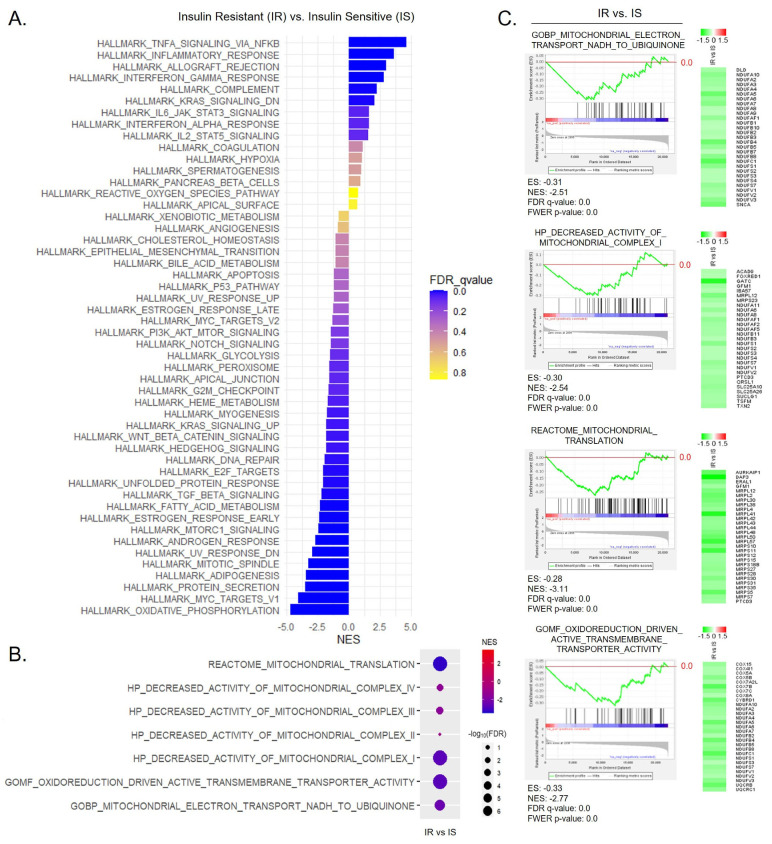
Comparison analysis of the insulin-resistant group vs. the insulin-sensitive group in omental adipose tissues from morbidly obese subjects. (**A**) Hallmark gene set analysis of the omental adipose tissue from insulin-resistant obese subjects (IR) compared to insulin-sensitive (IS) obese subjects. For enrichment analysis, fifty hallmark gene sets from the MSigDB were employed. Enrichment was significant for bars in purple with FDR < 25%, whereas bars in yellow indicate FDR > 25%. FDR *q*-values < 0.05 were considered as significant. The x-axis represents the NES, while the y-axis displays the fifty hallmark pathways arranged in descending order of NES scores, from the highest positive to the lowest negative. The color of the bar indicates the significance based on FDR *q*-value from blue to yellow, where lowest has vivid blue color with higher significance. (**B**) A dot plot illustrating significantly enriched gene sets related to the activity of mitochondrial complexes and regulation of the mitochondrial translation process. The size of the dot indicates significance, as determined by −log10(FDR *q*-value), and the color of the dot indicates NES. (**C**) Enrichment plots of gene sets related to insulin receptor signaling pathway, decreased activity of mitochondrial complex I, mitochondrial electron transport from NADH to ubiquinone, and mitochondrial translation, derived from GOBP, HP, and Reactome. A heatmap illustrates the expression levels of highly enriched genes within each gene set.

To confirm the interaction between dysregulated mitochondrial energy expenditure and mitochondrial translation in relation to insulin resistance in humans, we utilized gene expression data from insulin-resistant and insulin-sensitive obese subjects obtained from the GEO database. A comprehensive explanation of the experimental methodology utilized for this study is provided in the Materials and Methods section. To gain insights into the overall expression pattern, we conducted a 50 hallmark gene set analysis using GSEA, as depicted in Figure 7A. The bar length in the graph corresponds to the strength of the enrichment, whether positive or negative. The color gradient ranges from blue to yellow, with vivid blue representing high significance and lower *q*-values, gradually transitioning to yellow. In the gene expression analysis of insulin-resistant patients, we observed a significant enrichment of gene sets associated with inflammatory responses, including TNF-α signaling via NF-κB, allograft rejection, and IFN-γ response. Conversely, we found negatively enriched gene sets associated with lipid metabolism, such as oxidative phosphorylation, as indicated by the GSEA hallmark expression pattern. Supplemental Figure S6A illustrates the top enriched pathways, with the TNF-α signaling via NF-κB gene set demonstrating the highest positive enrichment, while the oxidative phosphorylation gene set exhibited the most significant negative enrichment. Supplemental Figure S6B presents a dot plot illustrating the increased activation of inflammatory responses in the insulin-resistant group compared to the control group.

Consistent with the expression profiles observed in mice, we also observed a significant and negative enrichment of gene sets associated with mitochondrial energy expenditure and mitochondrial translation (Figure 7B,C). The dot plot in Figure 7B demonstrates the specific downregulation of mitochondrial electron transfer from NADH to ubiquinone in complex I in the insulin-resistant group, along with the suppression of mitochondrial translation. Detailed enrichment plots with representative genes are listed in Figure 7C. To validate the overlap between enriched genes in each gene set, we listed the numbers of overlapped genes in the Venn diagram displayed in Supplemental Figure S6C. This diagram illustrates the shared genes between the gene sets associated with mitochondrial energy expenditure and mitochondrial translation. As a note, no overlapped enriched genes were detected between the mitochondrial electron transport from NADH to ubiquinone and mitochondrial translation gene sets.

## 4. Discussion

Our recent publication presents compelling evidence highlighting the significance of liver inflammation in the context of white adipose tissue inflammation in mice fed with an HFD [18]. Specifically, we found that the metabolic disruption in mitochondria, specifically focusing on NADH homeostasis, within the hepatic system played a pivotal role in intensifying inflammation in eWAT and perpetuating a state of systemic inflammation. These findings underscore the critical interplay between liver inflammation and metabolic dysregulation, shedding light on the mechanisms that drive eWAT inflammation and its systemic consequences in the context of obesity. Based on these research findings, here, we present the potential mechanisms underlying the beneficial effects of allulose in insulin resistance and metabolic disorders, highlighting the inhibitory mechanisms of allulose on HFD-induced chronic inflammation via the suppression of early hepatic inflammation. In the following sections, we will carefully examine the implications of our findings, delving into their potential significance in the field of insulin resistance and metabolic disorders.

Using hallmark gene set signatures and targeted analysis, our study demonstrated that allulose treatment effectively enhanced IFN-γ signaling and alleviated macrophage dysfunction in the liver and eWAT of HFD-fed mice. This finding emphasizes the crucial role of this mechanism in the progression of chronic inflammation associated with obesity. It aligns with previous reports and underscores the significance of IFN-γ signaling and dysregulated macrophage function in the context of chronic inflammation induced by an HFD [24,25]. Through a comparison of hepatic gene expression between the HFD-fed mice and a mouse model with a chronic expression of IFN-γ, achieved by the deletion of AU-rich elements (AREs) in the 3′-untranslated region (UTR) of IFN-γ, we identified macrophage dysfunction as a key contributor to HFD-induced chronic inflammation and validated these findings in human obese subjects using data from genome-wide association studies (GWAS) [24]. Furthermore, we provided initial evidence indicating that a prolonged expression of IFN-γ leads to impaired autophagy function in macrophages [25], thus highlighting the involvement of IFN-γ in the induction of macrophage dysfunction.

Although it is well known that macrophages play a crucial role in obesity-induced inflammation and insulin resistance, which are the key features of T2D [29,30], the specific mechanisms underlying their involvement in these processes remain to be fully elucidated. Some studies suggest that macrophage polarization, specifically the shift towards the pro-inflammatory M1 phenotype, plays a pivotal role in obesity-induced inflammation and insulin resistance, while the anti-inflammatory M2 phenotype has been associated with improved metabolic outcomes [31]. Our findings demonstrated that alterations in metabolic and inflammatory responses were associated with the M1 phenotype, characterized by suppressed oxidative phosphorylation, mitochondrial beta-oxidation, and heightened signaling pathways of TNF-α and IL-6. However, our observations indicate that allulose treatment effectively suppresses the enhanced IL-10 signaling pathway induced by an HFD, suggesting that the suppressive effect of allulose on M1 macrophages is not primarily through M2 polarization but rather through the direct suppression of M1 macrophages themselves. Interestingly, the pathway analysis of enriched genes from gene sets involved in macrophage dysfunction indicates that IFN-γ is the key factor involved in dysregulating macrophage function, and this pathway is strongly suppressed by allulose treatment. Therefore, these findings provide additional support for the hypothesis that allulose directly targets and suppresses M1 macrophages via the suppression of IFN-γ.

Mitochondrial dysfunction, which is characterized by reduced mitochondrial energy expenditure and impaired oxidative phosphorylation, has been implicated in the development of insulin resistance and T2D [32,33,34]. Our analysis of gene expression profiles in PBMCs from T2D patients indicates that the mitochondrial energy expenditure pathway and mitochondrial translation process can be recovered by allulose treatment and that these two processes are closely correlated. It also implies that these pathways can affect immune cell dysfunction such as macrophages. Furthermore, our findings specify that the mRNA translation mediated by RBPs, particularly IGF2BPs, contributes to the development of HFD-induced T2D. Recent studies have highlighted the significant roles of mitochondrial translation and RBPs in the development of insulin resistance and T2D [35,36,37,38]. Our data provide valuable insights into the interplay between these mechanisms, underscoring their interconnectedness. These findings emphasize the potential of targeting these pathways as a therapeutic intervention for metabolic disorders.

To elucidate the intricate mechanisms underlying mitochondrial metabolic dysfunction, we conducted a targeted analysis with a specific focus on the mitochondrial energy expenditure process. Our results, although not fully presented here, consistently supported the notion that gene sets associated with mitochondrial fatty acid beta-oxidation, the tricarboxylic acid (TCA) cycle, and acyltransferase activity were suppressed or dysregulated in response to HFD, and their functionality was restored by the allulose treatment. Based on the literature, these pathways play a role in the generation of NADH, the reduced form of nicotinamide adenine dinucleotide (NAD), and the level of NADH regulates the activity of these pathways [39]. Moreover, our data demonstrated that electron transport from NADH to ubiquinone in complex I of the electron transport chain (ETC) was particularly inhibited by the HFD, implying an increased NADH level in mitochondria. Therefore, it is reasonable to suggest that the suppression of these pathways observed in our study was likely due to the increased levels of NADH.

Elevated NADH levels resulting from impaired mitochondrial function and disrupted metabolic pathways are associated with an imbalance in the NAD+/NADH ratio, a common occurrence in insulin resistance, obesity, and T2D [40,41,42]. Based on our data, this mechanism plays a pivotal role in HFD-induced insulin resistance. To gain deeper insights into this process, we conducted further investigations in human subjects by analyzing gene expression profiles in omental adipose tissue. Specifically, we compared obese individuals with insulin resistance to a group of individuals who exhibited insulin sensitivity. By examining the differences between these two groups, we aimed to unravel the specific relationship between the pathway under investigation and the development of insulin resistance in the context of obesity. The hallmark gene set signature demonstrated that increased inflammatory responses such as IFN-γ and TNF-α, along with a reduction in oxidative phosphorylation, were strongly detectable in the insulin-resistant group. Consistent with our observation in HFD-fed mice, a significant suppression of mitochondrial electron transport from NADH to ubiquinone was detectable in the insulin-resistant group, accompanied by decreased mitochondrial translation. These findings shed light on the molecular characteristics associated with insulin resistance, indicating a disrupted metabolic state characterized by heightened inflammation, impaired oxidative phosphorylation, and suppressed mitochondrial electron transport. These alterations further disturb the delicate balance between NAD+ and NADH, contributing to the development of insulin resistance in T2D.

From a technical perspective, integrating biological data from different time points requires careful consideration of both experimental methodologies and physiological factors. Relying solely on temporal and dosage parameters for merging is insufficient. For example, although the composition of the HFD was similar between GSE137365 and GSE39549, we observed variations in the manifestation of identical physiological phenotypes across different time intervals. These variations can be attributed to factors such as daily caloric restriction, the number of mice housed per cage, and periodic blood sampling, all of which contributed to the overall stress levels experienced by the mice. Therefore, to properly integrate the data, it is crucial to have a comprehensive understanding of the experimental conditions and compare the physiological outcomes accordingly.

## 5. Conclusions

In summary, our study investigated the beneficial effects of allulose supplementation in insulin resistance and metabolic disorders. We found that the allulose treatment suppressed IFN-γ signaling and improved macrophage dysfunction in the liver and adipose tissue, mitigating obesity-induced chronic inflammation. Based on our prior report, these findings suggest that early liver inflammation plays a crucial role in initiating adipose tissue inflammation. Additionally, the allulose treatment restored mitochondrial energy expenditure and mitochondrial translation, which may be crucial in altering the immune response of immune cells, including macrophages. Our findings also highlight the interconnectedness of mitochondrial NADH homeostasis and mitochondrial translation in insulin resistance. The analysis of gene expression data from obese patients further supported these insights. Overall, our study enhances our understanding of the mechanisms underlying the beneficial effects of allulose in metabolic disorders and provides valuable insights into obesity-induced insulin resistance and T2D development.

## Data Availability

Not applicable.

## Supplementary Materials

The following supporting information can be downloaded at: https://www.mdpi.com/article/10.3390/nu15194218/s1, Figure S1: Hallmark gene set analysis in eWAT from mice fed HFD with or without allulose treatment; Figure S2: Effects of allulose treatment on inflammatory responses and lipid metabolism in eWAT of HFD-fed mice; Figure S3: Effects of allulose treatment on chemokine receptor signaling in eWAT of HFD-fed mice; Figure S4: Pathway analysis of enriched genes from the gene sets of impaired macrophage phagocytosis, impaired antigen-specific response, abnormal MHCII surface expression, and impaired oxidative burst in Figure 4B; Figure S5: Suppression of mitochondrial energy expenditure and mRNA translation gene sets in eWAT in response to HFD and its reversal upon allulose treatment; Figure S6: Enrichment plots of the most negatively and positively enriched pathways in Figure 7A.
