# Supplementary material for: D-Allulose Ameliorates Dysregulated Macrophage Function and Mitochondrial NADH Homeostasis, Mitigating Obesity-Induced Insulin Resistance
Source: Nutrients. 2023 Sep 29;15(19):4218. doi: 10.3390/nu15194218 (PMC10574141; doi:10.3390/nu15194218)
Supplement: Supplementary file 1 [file nutrients-15-04218-s001.zip › nutrients-2623507-supplementary.pdf]

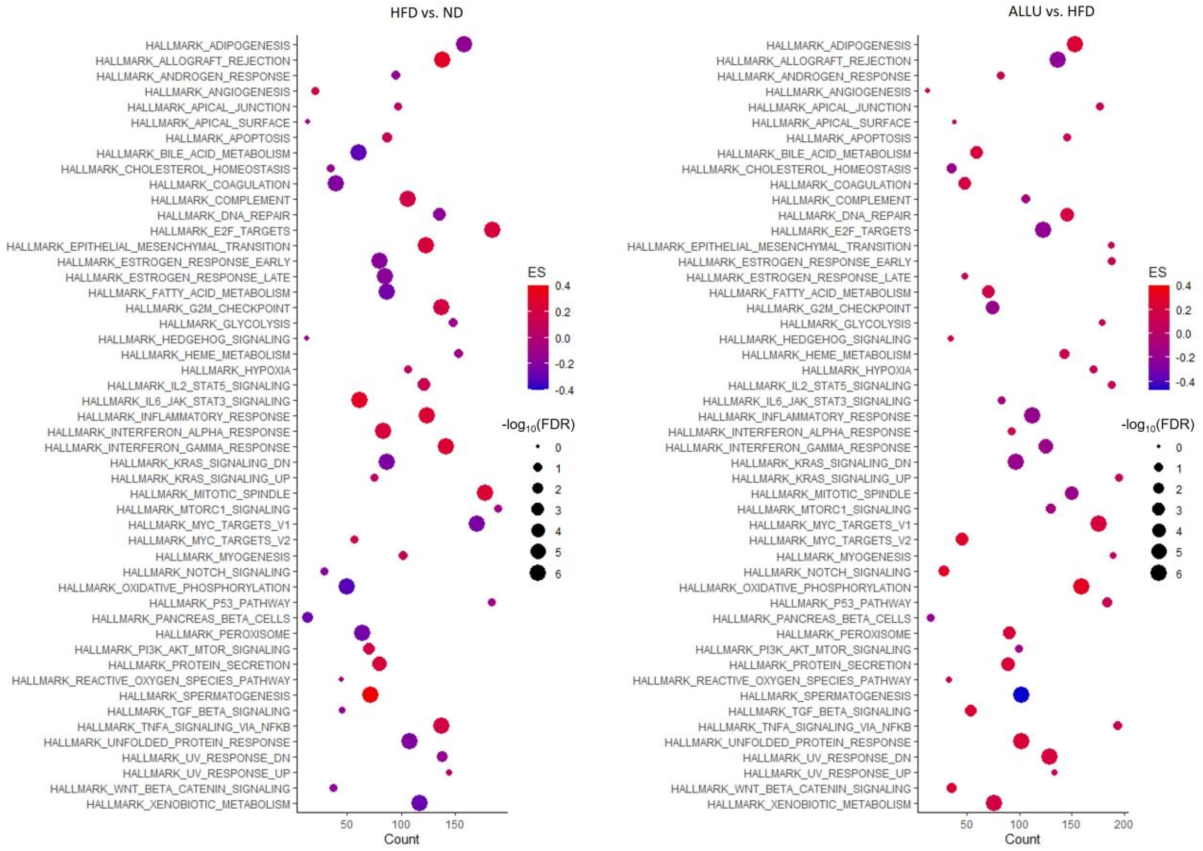

Supplemental Figure S1. Hallmark gene set analysis in eWAT from mice fed HFD with or without allulose treatment. Fifty hallmark gene set from the MSigDB were used for enrichment analysis. The x-axis (count) represents the number of significantly enriched genes in each pathway, determined using the GSEA algorithm. The y-axis displays fifty hallmark pathways. The dot size reflects the significance level, represented by the negative logarithm of the FDR q-value, while the dot color indicates the ES. Panel (A) presents a dot plot of 50 hallmark gene set analysis of the response to HFD, while panel (B) represents a dot plot of 50 hallmark gene set analysis of the response to HFD with allulose treatment. The baseline (0.0) of the enrichment score is indicated with a red color line.

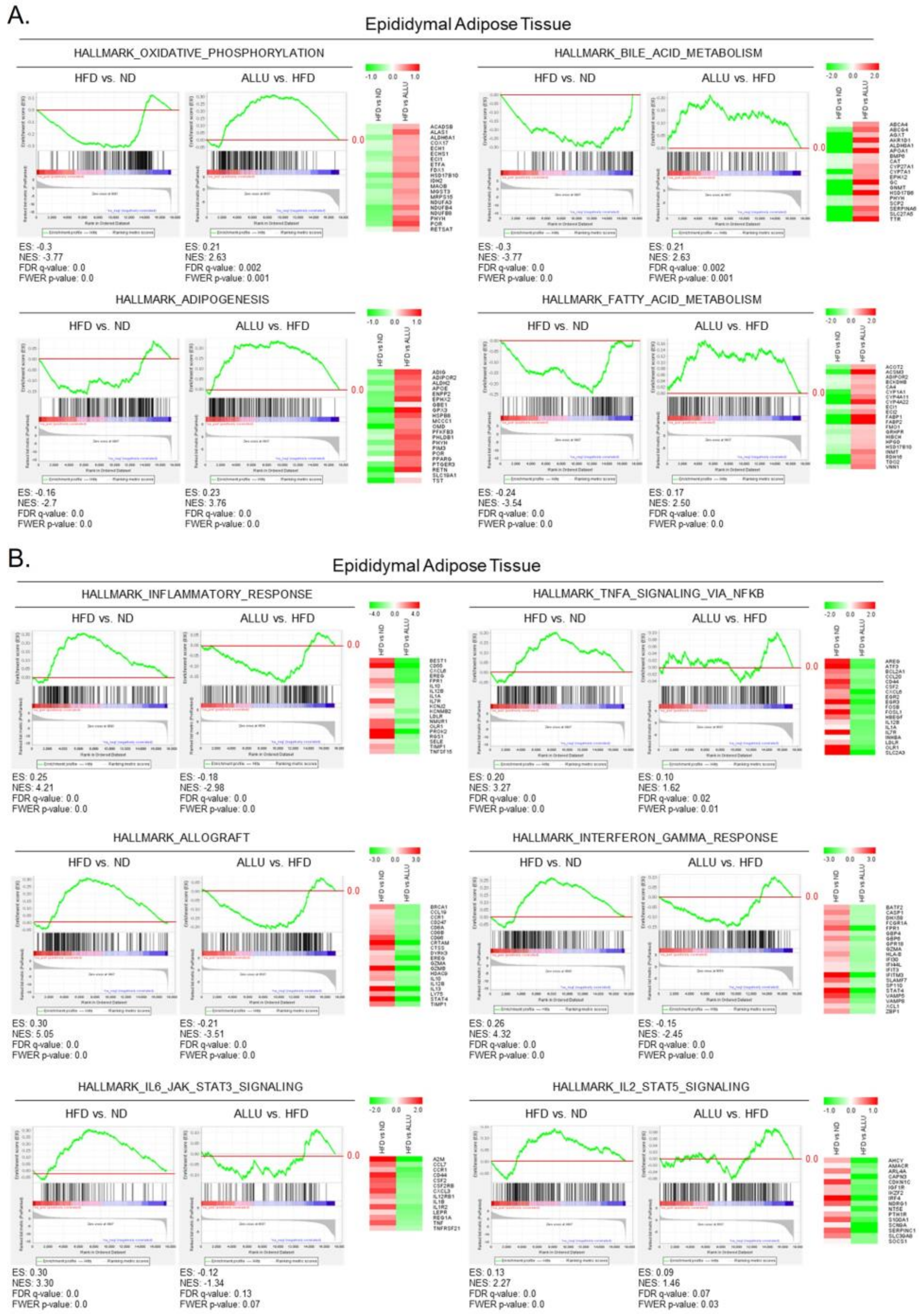

Supplemental Figure S2. Effects of allulose treatment on inflammatory responses and lipid metabolism in eWAT of HFD-fed mice. (A) Enriched plots generated from GSEA showing suppression of lipid metabolism in eWAT from HFD-fed mice and its reversal upon allulose (B) Enriched plots generated from GSEA showing induction of inflammatory responses in eWAT from mice fed the HFD and its reversal upon allulose treatment. A heatmap illustrates the expression levels of the representative enriched genes in each gene set.

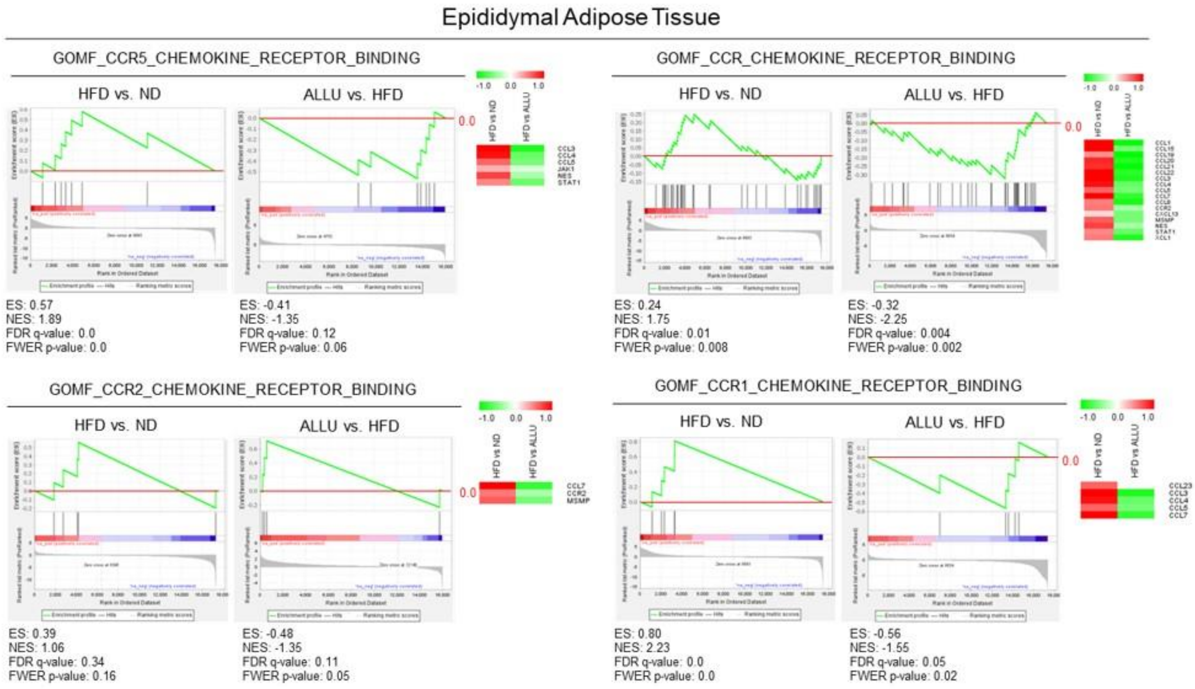

Supplemental Figure S3. Effects of allulose treatment on chemokine receptor signaling in eWAT of HFD-fed mice. Positively enriched plots generated from GSEA in response to HFD and its reversal upon allulose treatment with gene sets of CCR1, CCR2, CCR5, and overall CCR chemokine receptor binding from the MSigDB. A heatmap illustrates the expression levels of the representative enriched genes in each gene set.

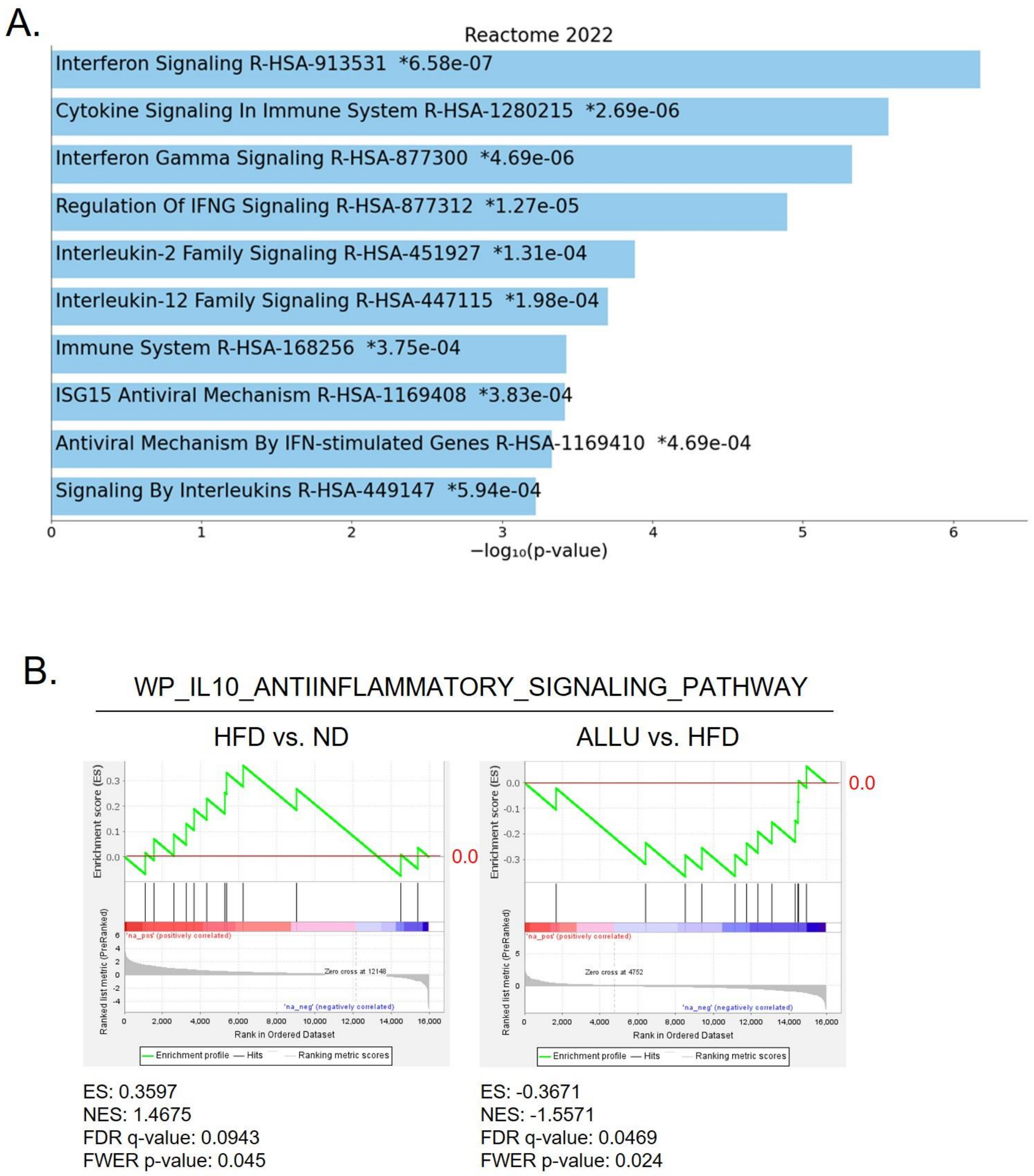

Supplemental Figure S4. (A) Pathway analysis of enriched genes from the gene sets of impaired macrophage phagocytosis, impaired antigen-specific response, abnormal MHCII surface expression, and impaired oxidative burst in Figure 4B. The Enrichr pathway analysis shows the top 10 pathways with opposite regulation between HFD and allulose treatment groups in (A) based on Reactome 2022 database. The x-axis represents the significance based on -log10(p-value), with the top pathways listed in descending order. (B) A enriched plot generated from GSEA in response to HFD and its reversal upon allulose treatment in HFD-fed mice liver with a gene set of IL-10 anti-inflammatory signaling pathway derived from WikiPathways (WP).

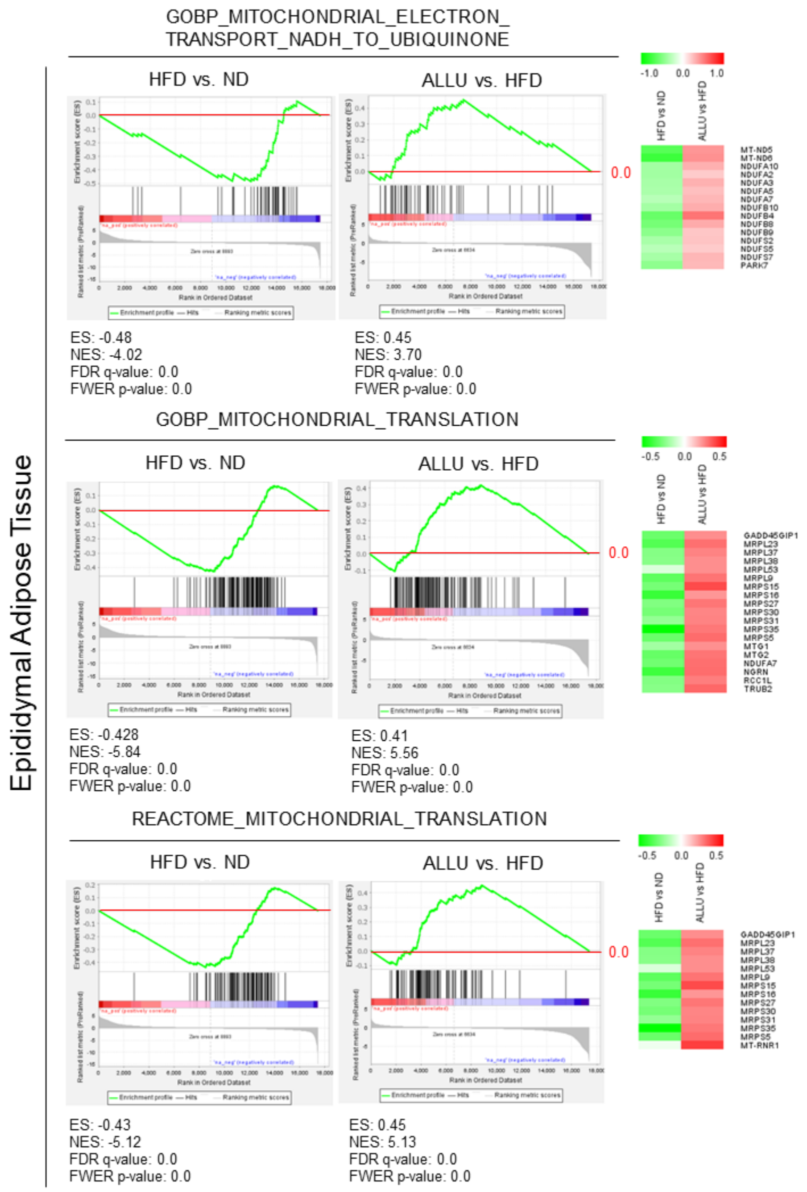

Supplemental Figure S5. Suppression of mitochondrial energy expenditure and mRNA translation gene sets in eWAT in response to HFD and its reversal upon allulose treatment. A heatmap illustrates the expression levels of representative enriched genes in each gene set.

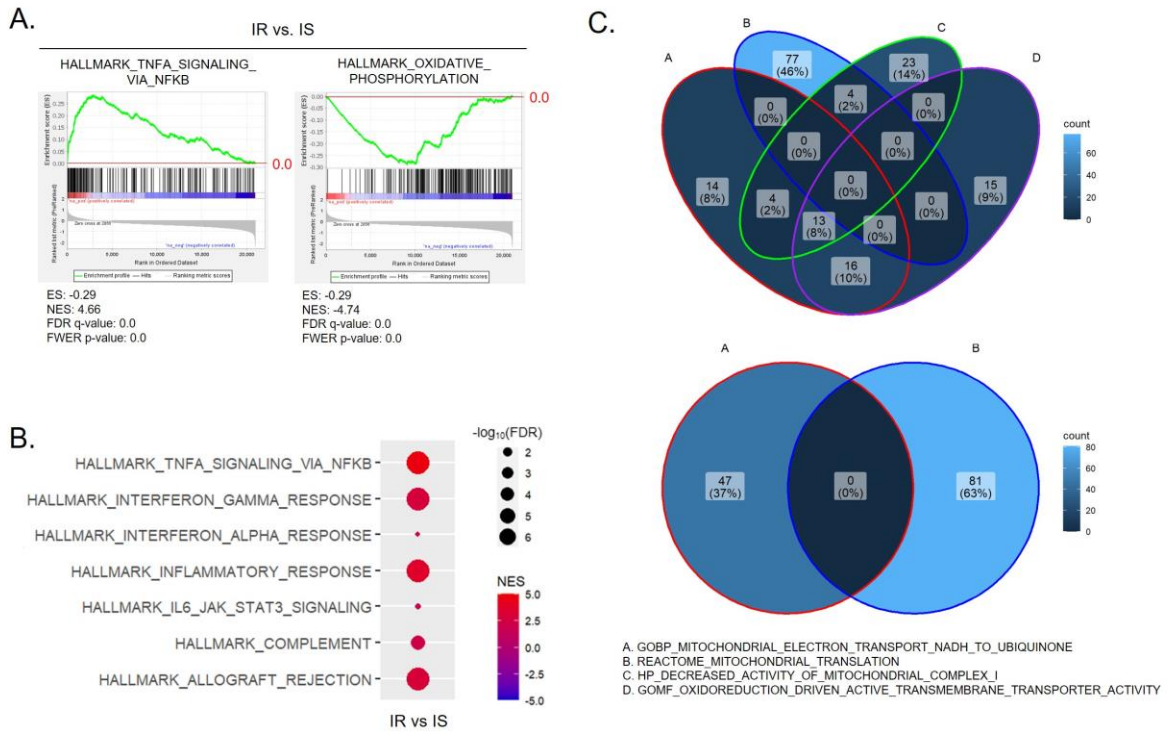

Supplemental Figure S6. (A) Enrichment plots of the most negatively and positively enriched pathways in A, which were oxidative phosphorylation and TNF-α signaling via NF-kB, respectively. (B) A dot plot of significantly enriched hallmark gene sets involved in the inflammatory responses. (C) Venn diagrams of overlapped gene numbers between gene sets in Figure 7C.
